# Laser Ablation for Small Hepatocellular Carcinoma

**DOI:** 10.1155/2011/595627

**Published:** 2011-12-04

**Authors:** Claudio Maurizio Pacella, Giampiero Francica, Giovanni Giuseppe Di Costanzo

**Affiliations:** ^1^Department of Radiology and Diagnostic Imaging, Regina Apostolorum Hospital, Albano Laziale, 00041 Rome, Italy; ^2^Ultrasound Unit Gastroenterology Department, S. Maria della Pietà Hospital, Casoria, 80026 Naples, Italy; ^3^Hepatology Unit, A. Cardarelli Hospital, 80131 Naples, Italy

## Abstract

Hepatocellular carcinoma (HCC) is one of the most common malignancies worldwide and is increasingly detected at small size (<5 cm) owing to surveillance programmes in high-risk patients. For these cases, curative therapies such as resection, liver transplantation, or percutaneous ablation have been proposed. When surgical options are precluded, image-guided tumor ablation is recommended as the most appropriate therapeutic choice in terms of tumor local control, safety, and improvement in survival. Laser ablation (LA) represents one of currently available loco-ablative techniques: light is delivered via flexible quartz fibers of diameter from 300 to 600 **μ**m inserted into tumor lesion through either fine needles (21g Chiba needles) or large-bore catheters. The thermal destruction of tissue is achieved through conversion of absorbed light (usually infrared) into heat. A range of different imaging modalities have been used to guide percutaneous laser ablation, but ultrasound and magnetic resonance imaging are most widely employed, according to local experience and resource availability. Available clinical data suggest that LA is highly effective in terms of tumoricidal capability with an excellent safety profile; the best results in terms of long-term survival are obtained in early HCC so that LA can be proposed not only in unresectable cases but, not differently from radiofrequency ablation, also as the first-line treatment.

## 1. Introduction

Hepatocellular carcinoma (HCC) is the sixth most common malignancy worldwide and the third leading cause of cancer-related death [1]. Its incidence is continually rising mostly as a result of the spread of hepatitis C virus infection [2–4]. Patients with chronic hepatitis B or C virus infection are at high risk of developing HCC. The annual incidence of HCC in hepatitis B virus infection carriers is approximately 0.5–1% and reaches 2.5% in the presence of liver cirrhosis [5, 6]. The incidence of HCC in chronic hepatitis C-related liver cirrhosis is up to 2–8% [6–8]. With surveillance of the high-risk population by ultrasonography and *α*-fetoprotein (AFP), HCC is increasingly detected at small size, that is, 5 cm or smaller in diameter [9–11]. However, a careful multidisciplinary assessment of tumor characteristics, liver function, and physical status is required for proper therapeutic management even in patients with early-stage tumors [12]. Approximately, 30% to 40% of all HCC patients have early tumors which can be treated with curative therapies such as resection, liver transplantation, or percutaneous ablation [12]. While surgical resection provides favourable long-term survival [13, 14], only a minority of patients are suitable for resection owing to multiple tumor nodules, concomitant decompensated liver cirrhosis, lesion location, and/or severe portal hypertension. Similarly, surgical resection carries a significant associated morbidity and mortality as well as a disease recurrence rate of up to 75% [15, 16]. Liver transplantation is more indicated in small HCC associated with severe cirrhosis and offers a satisfactory long-term outcome [16–18], but the shortage of donor organs significantly limits its application. When surgical options are precluded, image-guided tumor ablation is recommended as the most appropriate therapeutic choice and is considered a potentially radical treatment in properly selected candidates [12]. This technique is also cost effective in comparison to other treatments and is able to maximize the preservation of surrounding liver parenchyma whilst reducing in-patient hospitalization rates [12, 19, 20]. However, whether or not percutaneous ablation can compete with surgery as the first-line treatment remains highly controversial and subject of debate. Laser ablation (LA) represents one of a range of currently available loco-ablative techniques with the majority of reported data coming from Italy, Germany, and the UK [21].

## 2. General Principles and Techniques

### 2.1. Image-Guided Tumor Ablation

The term “image-guided tumor ablation” is defined as the direct application of chemical or thermal therapies to a specific focal tumor(s) in an attempt to achieve eradication or substantial tumor destruction [22]. Over the past 25 years, several methods for chemical or thermal tumor destruction have been developed and clinically tested [23], and image guidance is critical to the success of these therapies [21, 24]. Although tumor ablation procedures can be performed at laparoscopy or surgery, most procedures treating focal lesions are performed with percutaneous approach, explaining why these procedures are often referred to as “percutaneous therapies.”

 Thermal energy sources such as laser, radiofrequency (RF), or microwave (MW) make the destruction of tumors possible without surgical removal. The potential advantages of in situ tumor ablation include decreased costs, reduced morbidity, the possibility of performing procedures on outpatients, and the possibility of treating patients who are poor candidates for surgery due to age, comorbidity, or extent of disease. The thermal ablation therapies involved in clinical practice can be classified as either hyperthermic treatments, including RF ablation (RFA), laser ablation (LA), MW ablation (MWA), and high-intensity focused ultrasound (HI-FU) or cryoablation (CA). The thermal damage caused by heating depends on both the tissue temperature reached and on the duration of heating. On the other hand, freezing tissue at temperatures between −20°C and −60°C, followed by rapid thawing, results in cell membrane disruption and induces cell death. In order to adequately destroy tumor tissue, the entire target volume must be subjected to cytotoxic temperatures [25].

### 2.2. Laser Ablation

The term “laser ablation” refers to the thermal destruction of tissue by conversion of absorbed light (usually infrared) into heat and includes various technical variations on this theme including “interstitial photocoagulation”, “laser coagulation therapy”, “laser interstitial tumor therapy”, and “laser-induced thermal therapy” [26–34]. Neodymiun:yttrium aluminum garnet (Nd:YAG) lasers with a wavelength of 1064 nm are usually used for percutaneous LA because penetration of light is optimal in the near infrared spectrum [35]. In recent years, diode lasers with suitable wavelengths have also been used [36]. Laser light penetrates directly for a distance of between 12–15 mm as a result of backward and forward scattering and reflection and absorption. Scattering results in a relatively uniform distribution of absorbed energy, and heat is produced by conversion of absorbed light. Further away from the point of application, the tissue is heated by conduction creating large coagulative zones [27, 29, 30]. Temperature above 60°C results in a rapid coagulation zone and instant cell death. Irreversible cell death, without preceding coagulation, occurs also at lower hyperthermic temperatures (>42°C) but requires durations of treatment that vary negatively with temperature [29, 30, 37, 38]. Thus, it has been shown that LA at 46°C requires treatment for 30 minutes to ensure radicality [29, 39]. Temperatures above 100°C will cause vaporization from evaporation of tissue water, and above 300°C, tissue carbonization occurs. Overheating is thus best avoided as carbonization decreases optical penetration and heat conduction and limits the size of lesions produced [33].

 A high optical penetration depth reduces the temperature gradient between the laser fiber and tissue and lessens the risk of overheating which prevents carbonization and vaporization of tissue [33]. The optical penetration depth is greater in metastatic tissue than in normal liver, being 4.2 and 3.0 mm, respectively, at the 1064 wavelength. Coagulation necrosis reduces optical penetration by about 20% in both normal and tumor tissue [33, 40].

 Using a bare tip, almost spherical lesions with a maximum diameter of 12–16 mm can be produced [21]. Lesion size can be increased by using beam splitters for simultaneous use of multiple fibers in an array around the tumor [32, 41]. This avoids the problem of repositioning single fibers and the difficulties associated with treatment-induced imaging artifacts. In vitro studies have shown that simultaneous treatment with four fibers at 2.0 cm separation produces lesions that are 11 times bigger than those produced with a single fiber, creating a lesion cylinder 4.0 cm in diameter and 3 cm in height [42]. Simultaneous multifiber treatment produces lesions averaging 3.6 × 3.1 × 2.8 cm in normal canine liver [41]. Bare fibers are now being replaced more and more often by specially designed fibers equipped with diffuser endings that emit light up to a distance of 10–30 mm and with liquid cooling that increases lesion size by allowing higher power while avoiding charring [43, 44]. By simultaneously inserting four fibers and using fiber cooling, tumors with a diameter of 5.0 cm can be safely eradicated in a single treatment session, as reported in the largest series of local ablation in a single institution [45]. Recently, using a 980 nm diode laser ablation system in an in vivo tumor model with an internally cooled applicator, large ellipsoid thermal ablation with sharp boundaries was obtained in less than 3 minutes [36].

Local tissue properties, in particular perfusion, have a significant impact on the size of ablative zone. Highly perfused tissue and large vessels act as a heat sink, as laser light is absorbed by erythrocytic heme and transported from the local area [46]. This phenomenon makes native liver parenchyma relatively more resistant to LA than tumor tissue and is the basis for the use of hepatic inflow occlusion techniques in conjunction with laser therapy such as local arterial embolization [47–51].

 One advantage of using laser technology is that there are sophisticated systems for controlling ablative lesion characteristics and size using feedback systems, and dose-planning systems are available with laser technology [39, 42, 52–54]. An important practical advantage over other thermal sources is that the thin and flexible laser fibers make it possible to reach tumors more easily and safely [55].

 Light is delivered via flexible quartz fibers with a diameter from 300 to 600 *μ*m. Conventional bare tip fibers provide a near spherical lesion about 15 mm diameter at their ends but have been largely replaced by interstitial fibers, which have flat or cylindrical diffusing tips and are 10–40 mm long, providing a much large ablative area of up to 50 mm [56, 57]. The use of beam splitting devices allows for the use of up to four fibers at once with corresponding increase in ablative volume: multiple fibers must be placed, however, and the devices only work effectively at lower powers [55]. By increasing laser power, light transmission improves and the ablative zones increase in size. However, increasing laser power also causes local temperature to rise, leading to the risk of overheating and carbonization of adjacent tissue. The use of water-cooled laser application sheaths allows a higher laser power output (up to 50 W compared with 5 W) while preventing carbonization [58]. Thus, the use of multiple water-cooled higher-power fibers allows ablative zones of up to 80 mm diameter [44]. Water-cooled sheaths do require wider-bore cannulas but are commonly placed via a coaxial dilatation system from an 18 G puncture. Recently, a novel ablation laser system consisting of a 15 W, 980 nm diode laser, flexible diffusing-tipped fiber optic, and 17-gauge internally cooled catheter was introduced in clinical practice; it achieves a large well-circumscribed ellipsoid ablation zone up to 42 mm in greatest diameter (with the lasers operating at 15 W for 120 seconds and two applicators) with sharp boundaries between thermally ablated tumor in the center surrounded by viable tumor tissue [36].

### 2.3. Real-Time Monitoring and Treatment Planning

Of the range of imaging modalities available to guide percutaneous laser ablation, the choice is based largely on local experience and resource availability. Local tumor destruction with heat is based on the assumption that the whole tumor can be selectively destroyed by raising tumor temperature to a critical level for a definite period of time. This can be ensured by a method that gives good thermal resolution of the relevant tissue or, preferably, accurate real-time monitoring of irreversible tissue damage. Non-invasive techniques are ideal for clinical thermometry, but none yet fulfills the requirements of routine clinical use.

 Ultrasound- (US-) guided needle placement has the advantage that it is quick, portable, and widely available; it is familiar also to those used to performing US-guided biopsies. The main disadvantage is that it offers little reliable indication of the temperature or actual extent of the ablative zone being created. In addition, US is not well suited because of treatment-induced artifacts [48, 59, 60]. CT is not useful for real-time monitoring because thermal tissue changes are not visible within 24 hours [61].

In contrast, magnetic-resonance- (MR-) guided LA, often in conjunction with liver specific contrast agent, offers real-time thermal mapping that allows the operator to visualize the size, location, and temperature of the ablation zone [58, 62]. This technique tends to be used in combination with the higher-power water-cooled laser systems as the increased energy can be delivered in a safe and controlled manner [63, 64]. However, MR-guided LA is limited by machine availability and the procedural complexity with an overall procedure time between 60 and 120 minutes [45, 65]. This may explain why laser ablation is not widely used compared with RF ablation. Newly developed MR-compatible applicators allow performance of the whole procedure in the MR suite, which may reduce procedure time and improve technical effectiveness. This is under investigation in clinical trials [66].

US-guided laser systems, instead, tend to use multiple lower-power fiber with a lower total energy delivery resulting in truly quick procedure time [55, 67]. It is possible to achieve a mean ablation zone diameter of 3.0 cm with this technique with a single illumination of 6 minutes [55].

Thermal imaging can be performed on most MR systems, with thermal changes being easier to demonstrate as magnet field strength increases. There are several methods of measuring tissue temperature changes with MR. The simplest, and most widely used technique, is measuring modifications in T1 value of tissues which decreases in linear relationships with increasing tissue temperature up to approximately 55°C [68]. Techniques that measure changes in the tissue diffusion are more accurate, up to ±1°C, but require long acquisition times and, therefore, suffer from patient motion artifacts [69]. Another technique measures changes in the proton resonance frequency shift (phase shift). Again, this measures temperature changes very accurately but is not suitable for use in fatty tissue and is less suited to open MR units as it requires a homogeneous magnetic field [70]. All of these techniques are best used in combination with subtraction techniques, that is, pretreatment image subtraction from heating image, which allows very accurate assessment of lesion size, but is sensitive to motion and misregistration artifacts [71].

 In addition to thermometry, the use of “open” magnets with the inherent lower field and gradient strengths allows real-time imaging of needle placement in a multiplanar manner, at variance with CT and is not limited by the presence of bone or gas, unlike US. The drawbacks of open magnets are the lower image quality and the increased scan time.

In contrast, conventional closed magnets require fiber placement using CT or US with subsequent transfer into MR scanner for thermal mapping. This has the disadvantage of requiring patient transfer mid procedure although it does provide faster imaging and thermal mapping than an open system [43, 67]. The use of MR guidance and thermometry is currently only feasible with LA systems, as it uses a completely metal free system and does not produce any radiofrequency interference [68, 69]. Most RF systems are currently not suitable for MR usage, both because of steel within the electrodes and the degradation of image quality by extraneous RF noise produced by the RF generators. Although MR-compatible systems have been developed in practice, the RF noise remains problematic [72].

 Considering the difficulties in real-time imaging, it is important that methods based on the production of heat ensure reproducible and cytotoxic temperatures in the periphery of tumor tissue. One way to ensure this is to use feedback control of the treatment effect, for example, to let measured temperatures control the energy output in order to achieve a constant temperature level and reproducible lesion size [39, 52]. Another way to control lesion size is to use a dose-planning system. This has been developed for LA and enables lesion size to be calculated for different tissues, output powers, and treatment duration [42, 73].

### 2.4. Patients

Selection criteria vary from unit to unit depending on the technique used and the facilities available [55, 74] but are broadly similar to those for other local ablative techniques and are based on size, number and site of HCC in patients who are deemed unsuitable for surgical resection or transplant. The ideal lesions are those of ≤3 cm in diameter, single or one to three nodules each ≤3 cm in diameter, irrespective of their location. Lesions adjacent to major vessels, biliary ducts, bowel, or diaphragm can be treated with caution even with MR-guided technique, but they are more safely approached with fine needle technique [55]. MR-guided technique allows confident ablation of anatomically more problematic lesions with the use of real-time thermometry and multiplanar MR targeting [74]. Severe liver disease (Child C) and coagulopathy are relative contraindication to be evaluated in each individual patient, and extrahepatic disease is generally considered an absolute contraindication. LA has also been proven safe and effective for the treatment of cirrhotic patients awaiting liver transplant surgery [75]. Large lesions ≥5 cm can be considered for laser ablation, particularly if using a high-power system, though they can require more than one treatment or/and pullback technique with fine needle method.

### 2.5. Outcome

LA has been performed mainly via the percutaneous route. The various laser fiber systems with their differing energy delivery levels make it difficult to compare their effectiveness and complication rates. In addition, the ranges of imaging modalities used at follow-up, combined with a variety of definitions of treatment success, make comparison of data difficult. Primary effectiveness relates to complete lesion ablation as assessed by either CT or MR at a defined point after procedure and after a defined number of treatments. Data regarding long-term survival rates for LA in part reflect the novelty of the treatment and the rapid advances in technology, particularly in relation to the laser fibers. LA has been proven safe and feasible for the treatment of HCCs in multiple cohort studies [45, 55, 63, 64, 67, 74, 76–79]. The results are summarized in Table 1. According to the multicenter study involving 432 cirrhotic patients (single tumor ≤4 cm or three nodules ≤3 cm each) with 548 lesions, the ideal candidates for LA are younger (<73 years) patients with normal serum albumin levels and tumor size ≤2 cm who are highly likely to achieve complete tumor ablation [79]. In this study, patients with Child-Turcotte-Pugh class A with tumor size ≤3 cm and a well-differentiated histologic pattern who achieved an initial complete ablation had a median survival time of 65 months (95% CL, 38 to 92 months), and those with a main tumor size ≤2 cm and sustained necrosis to initial complete response had a median survival time of 63 months (95% CL, 48 to 78 months), which further increased to 68 months (95% CL, 22 to 114 months) in those with a well-differentiated histology. The median time to recurrence was 24 months (95% CL, 20 to 28 months), and the median disease-free survival time was 26 months (95% CL, 22 to 30 months) [79]. Results reported by those groups using water-cooled higher-power MR-guided laser ablation have been promising. Eichler et al. [74] reported mean survival rates of 4.4 years (95% CL, 3.6–5.2) in a series of 39 patients with 61 HCCs with complete ablation rate of 98%.

 Large lesions can be successfully treated by combining LA with transarterial chemoembolization (TACE). This modality is a safe and effective palliative treatment for large HCC. In the treatment of 30 large lesions (3.5–9.6 cm) using LA followed by TACE, it was possible to achieve 90% ablation rate with survival rates of 92%, 68%, and 40% at 1, 2, and 3 years without significant complications [80]. Ferrari et al. [81] supported this suggestion, demonstrating improved ablation rates and survival in patients with HCC >5 cm given combination treatment over LA alone.

### 2.6. Complications

Reported complication rates for laser ablation compare favorably to those of surgery. Arienti et al. [82] reported complication rates for 520 patients with 647 HCCs treated with 1064 laser sessions considering 90 factors for each record, including tumor characteristics. They report 0.8% deaths and 1.5% major complication rate. An earlier report by Vogl et al. [45] included 899 patients, of whom 42 had HCC, with 2520 lesions treated with 2132 laser sessions under CT/MR guidance, with 0.1% mortality and 1.8% major complication rate. Both groups reported common side effects of asymptomatic pleural effusion (7.3% and 6.9%), postprocedural fever (33.3% and 12.3%), and severe pain (7.5% and 11.5%). Neither of the above groups reported tumor seeding. As a rule, tumor seeding has rarely been reported after laser ablation.

### 2.7. Comparison with Other Hyperthermic Ablative Techniques

In their randomized controlled prospective (RCT) study, Ferrari et al. [83] treated 81 cirrhotic patients with 95 biopsy proven ≤4 cm HCCs comparing LA with RF ablation. Two matched groups were randomized to US-guided RF or LA under general anesthesia. The authors adopted multiple fiber technique using 5 W per fiber delivering a maximum of 1800 J per fiber per single illumination [55]. Assessment of response was evaluated by dynamic CT scan. They reported no significant difference overall in survival rates between the two methods, with cumulative rates of 91.8%, 59.0%, and 28.4% at 1, 3, and 5 years, respectively. However, they demonstrated a statistically significant higher survival rate for RF over LA for Child A patients (*P* = 0.9966) and nodules ≤2.5 cm (*P* = 0.01181). They reported no significant complications. This work added to the same groups' previous published improved survival rates in patients treated with LA, compared to those treated with either TACE or percutaneous ethanol injection (PEI) [84]. To date, Di Costanzo et al. (unpublished data) treated in an ongoing RCT (estimated sample, 140 patients) 95 patients with 109 biopsy proven HCCs (single tumor ≤5 cm or three nodules ≤3 cm each) with RF and LA (45 and 52, resp.). The two groups were comparable for median age (71 years), male/female (33/12-RF, 34/16-LA) Child-Turcotte-Pugh A/B (40/5-RF, 46/4-LA), and median diameter of HCC nodules (2.5 cm-RF, 3.0 cm-LA). With a median follow-up of 9 months (range, 1–25 months), complete tumor ablation (CTA) was observed in 95.5% and 96.0% of cases in RF and LA, respectively. Overall, HCC recurrence occurred in 24 (15 RF and 9 LA) patients. Local recurrences were 9 in RF group and 3 in LA group. Median time tumor recurrence (TTR) was 15 (95% CL, 10–20) and 19 (95% CL, 17–23) months in RF and LA groups, respectively, (*P* = 0.051). One-year progression-free survival rate was 55% in RF group and 74% in LA group. One-year survival probability was 92% and 95% in RF and LA groups, respectively. Neither major complications nor significant treatment-related morbidity was registered in either groups. Di Costanzo et al. concluded that LA was as effective as RF in inducing the complete response of HCC nodules. A longer TTR and a lower recurrence rate were observed in LA group. These interesting data have to be evaluated and confirmed at the end of the follow-up anticipated in June 2013.

## 3. Conclusion

LA of HCC has proven itself to be a safe and effective local therapy for patients with HCC nodules for whom surgical resection is not possible. Nevertheless, further data from randomized trials are required to establish long-term survival rates for higher-power water-cooled systems before this kind of treatment can be fully validated as a standard treatment. It appears that RF, LA, or MW should replace PEI and cryotherapy, and, to date, there is little difference in outcome between RF and LA using the latest technological developments [21, 85].

 Thus far, local ablation of hepatic disease has been used for unresectable tumors, but these hyperthermic therapies might be considered as a first-line therapeutic option in the subgroup of patients with HCC nodules ≤2cm in diameter [86, 87] and, in our opinion, particularly in cases with well-differentiated histologic pattern [79]. In the future, with the refinements of the technology and increased experience with these techniques, it will be possible to safely and completely destroy HCC ≥5 cm as well, even in patients suitable for surgery. The advantage of local tumor destruction include (a) selective damage with a smaller immunosuppression and a smaller release of growth factors [21, 88], (b) minimal treatment morbidity and mortality, and (c) the possibility of starting chemotherapy before or at the time of local therapy [89, 90]. The main problems with in situ ablation are the lack of good imaging techniques to determine the actual extent of disease at intra- and extrahepatic sites and the lack of real-time monitoring of treatment precision and irreversible tissue effect. The percutaneous approach needs to be developed and has not yet been proven to give complete tumor ablation rates that permit its use outside controlled clinical studies. It might be valuable in a few truly unresectable or inoperable patients or in selected patients. In the vast majority of cases, it should still be used and evaluated only in prospective randomized studies.

##  Conflict of Interests 

The authors declare that they are not shareholders and do not have any financial interests in the companies mentioned in this paper that compromised the design of research, the safety and wellbeing of patients, the collection and interpretation of research data, and as well as the dissemination of research results.

## Figures and Tables

**Table 1:** Studies reporting the outcome of Laser Ablation for small hepatocellular carcinoma.

Authors	Number of tumors/patients	Size of tumors (cm) mean (SD)	Local recurrence rate (%)	Complete ablation* (%)	Overall survival (%)		Major complications rate** (%)	Mortality rate (%)
					3-year	5-year		
Giorgio et al. [76]	85/77	3.2 (1.0–6.6)	18	82.5^+^			3.7^+^	1.2^+^
Pacella et al. [80]	30/30	5.2 ± 0.0 (3.5–9.0)	7	90.0^+^ TACE	60.0^§^		0	0
	15/30	1.9 ± 3.5 (0.8–3.0)	0	100.0			0	0
Pacella et al. [67]	92/74	2.4 ± 0.7 (0.8–4.0)	6	97.0	73.0^§^	31.0^§^	0	0
Eichler et al. [74]	39/61	>2.0	0	97.5	Mean 4.4 years		0	0
Dick et al. [64]	19/19			50.7^#^	Mean 14.6 months^#^			
Pacella et al. [77]	169/148	2.6 ± 0.8	15	82.0	58.0^§^	30.0^§^	0.5	0.6
Pacella et al. [79]	548/432 (multicenric study)	2.4 ± 0.8	20	79.6		41.0^§^	1.6	0.2

^+^Calculated in mix histologic tumor types (seventy-seven patients had hepatocellular carcinoma (HCC)). Twenty-seven had metastases from colocarcinoma (*n* = 25) or lung (*n* = 2).

*Calculated per tumor.

**Calculated per patient.

^§^Calculated in patients with Child-Turcotte-Pugh class A.

^#^Calculated in mix histologic tumor types (nineteen patients had 19 HCCs, eleven patients had metastases from a variety of primary tumors, and five patients had metastatic carcinoid).
